# Maternal and child factors associated with timely initiation of breastfeeding in sub-Saharan Africa

**DOI:** 10.1186/s13006-021-00402-3

**Published:** 2021-07-19

**Authors:** Francis Appiah, Bright Opoku Ahinkorah, Eugene Budu, Joseph Kojo Oduro, Francis Sambah, Linus Baatiema, Edward Kwabena Ameyaw, Abdul-Aziz Seidu

**Affiliations:** 1 Department of Population and Health, College of Humanities and Legal Studies, University of Cape Coast, Cape Coast, Ghana; 2 Berekum College of Education, Berekum, Bono Region Ghana; 3 Faculty of Health, School of Public Health, University of Technology Sydney, Ultimo, NSW Australia; 4 Department of Health, Physical Education, and Recreation, University of Cape Coast, Cape Coast, Ghana; 5 College of Public Health, Medical and Veterinary Sciences, James Cook University, Townsville, Queensland Australia; 6 Department of Estate Management, Takoradi Technical University, Takoradi, Ghana

**Keywords:** Maternal and child factors, Timely initiation of breastfeeding, SSA, DHS, Maternal health, Global health

## Abstract

**Background:**

The probability of not breastfeeding within the first hour after delivery (timely initiation of breastfeeding) is particularly pronounced in sub-Saharan Africa. In this study, we examined the maternal and child factors associated with timely initiation of breastfeeding in sub-Saharan Africa.

**Methods:**

We pooled data from 29 sub-Saharan African countries’ Demographic and Health Surveys conducted from 2010 to 2018. A total of 60,038 childbearing women were included. Frequencies, percentages, and binary logistic regression analyses were carried out. Binary logistic regression was used to examine the maternal and child factors associated with timely initiation of breastfeeding and the results were presented as adjusted odds ratios (aOR) at 95% confidence interval (CI).

**Results:**

We found a prevalence of 55.81% of timely initiation of breastfeeding in the sub-region. The country with the highest prevalence of timely initiation of breastfeeding was Burundi (86.19%), whereas Guinea had the lowest prevalence (15.17%). The likelihood of timely initiation of breastfeeding was lower among married women, compared to never married women (aOR 0.91; 95% CI 0.85, 0.98); working women compared to non-working women (aOR 0.90; 95% CI 0.87, 0.93); women who watched television at least once a week, compared to those who never watched television (aOR 0.74; 95% CI 0.70, 0.78); women who delivered through caesarean section, compared to vaginal birth (aOR 0.30; 95% CI 0.27, 0.32); and those with multiple births, compared to those with single births (aOR 0.67; 95% CI 0.59, 0.76). Women who lived in Central Africa were less likely to initiate breastfeeding timely compared to those who lived in West Africa (aOR 0.80; 95% CI 0.75, 0.84).

**Conclusions:**

The findings call for the need for a behavioural change communication programmes, targeted at timely initiation of breastfeeding, to reverse and close the timely initiation of breastfeeding gaps stratified by the maternal and child factors. Prioritising policies to enhance timely initiation of breastfeeding is needed, particularly among Cental African countries where timely initiation of breastfeeding remains a challenge. Sufficient supportive care, especially for mothers with multiple births and those who undergo caesarean section, is needed to resolve timely initiation of breastfeeding inequalities.

## Background

Timely initiation of breastfeeding has lifetime importance for the mother and the child [1, 2] and the Global Strategy for Infant and Young Child Feeding (GSIYF) sets the standards for timelyinitiation of breastfeeding [3]. Specifically, the GSIYF standards include 6 months of exclusive breastfeeding, and continued breastfeeding for 2 years or beyond with, timely, adequate, safe and appropriate complementary foods starting after 6 months. Related support for maternal health, nutrition and birth spacing is also important [3].

Globally, timely initiation of breastfeeding is low (44%) [4]. Newborns whose mothers do not initiate breastfeeding after the first hour of birth (33%) face a greater risk of common infections [5], have 50% increased risk of breathing difficulties in the first 6 months of life and increased risk of death compared with those whose mothers begin breastfeeding within 1 hour of birth [6, 7]. The risk of death increases with increasing delay in initiation of breastfeeding from 1 hour to day seven [5]. The first hour is important because it is within this period that newborns can be fed on colostrum (the first breast milk that provide passive and active protection against a wide variety of known pathogens) [8]. Thus, initiation of breastfeeding within the first 1 hour of delivery, is vital and can reduce neonatal mortality by 19.1% [9].

Studies have revealed a number of maternal and child factors as associated with timely initiation of breastfeeding. Ghimire [10] in a study among women of Nepal, indicated that educational level, ethnicity, mother’s involvement in agricultural occupations, and delivery in a health facility were associated with timely initiation of breastfeeding. Also, in Nepal, Adhikari [9] reported that the size of babies at birth and place of residence were associated with timely initiation of breastfeeding. Furthermore, Sigman-Grant and Kim [11] revealed that traditional beliefs and knowledge about specific breastfeeding are also linked to timely initiation of breastfeeding in Brisbane and Perth in Australia.

While some studies on timely initiation of breastfeeding in Africa have been published [1, 12–14], there is a dearth of studies linking maternal and child factors with timely initiation of breastfeeding across sub-Saharan Africa (SSA). Thus, we sought to examine the maternal and child factors associated with timely initiation of breastfeeding in SSA. Findings from the study may give a better understanding for policy interventions geared towards enhancing timely initiation of breastfeeding.

## Methods

### Data source

We pooled data from the most recent Demographic and Health Surveys (DHSs) of 29 countries in SSA from 2010 to 2018. Specifically, we used data from the child’s recode file of the various countries. The DHS is a nationally representative survey that has been conducted in over 85 low-and middle-income countries globally. It focuses on essential maternal and child health markers including “breastfeeding practices” [15]. The survey employs a two-stage stratified sampling technique, which makes the data nationally representative. The first-stage sampling consisted of a list of primary sampling units (PSUs) or enumeration areas (EAs) that covered the entire country and usually were obtained from the latest national census, when available. EAs are further subdivided into standard size segments of about 100–500 households per segment. In this stage, a sample of predetermined segments is selected randomly with probability proportional to the EA’s size (number of households in EA). In the second stage, the DHS personnel select households systematically from a list of previously enumerated households in each selected EA segment, and in-person interviews were conducted in selected households to target populations: women aged 15–49, men aged 15–64, and children under five. The women, those who were usual residents of selected households or visitors who slept in the households on the night before the survey are interviewed. Data on timely initiation of breastfeeding were obtained from the women through self-reporting. Aliaga and Ruilin, [16] provides details of the sampling process. A total of 60,038 women aged 15–49 years with birth history and who had children born in the 2 years preceding the survey and practiced breastfeeding were included in our study. We excluded women without birth history, those who did not give birth in the 2 years preceding the survey and those with no information on breastfeeding. As shown in Table 1, the overall number of missing values, was 2954, which represented 4.68% of the total sample. Due to the small proportion of missing values, we used complete cases for the analysis. We relied on the Strengthening the Reporting of Observational Studies in Epidemiology’ (STROBE) statement in writing the manuscript [17].

**Table 1:** Description of surveys and sample sizes

Country	Survey year	Sample^**a**^	Sample^**b**^	Sample^**c**^	Sample^**d**^
Angola	2015–16	14,322	3043	1402	1394
Burkina Faso	2017–18	15,044	2885	2972	2909
Benin	2010	13,407	4943	4417	4413
Burundi	2015–16	13,192	2591	2703	2609
Congo DR	2018	18,716	3704	3663	3452
Congo	2015	9329	2067	1064	1005
Cameroon	2012	11,732	2441	1568	1470
Ethiopia	2011–12	10,641	4016	4255	4096
Gabon	2013–14	6067	1682	235	212
Ghana	2016	5884	1218	1222	1171
Gambia	2012	8088	1677	1654	1596
Guinea	2013	7039	1447	1471	1429
Kenya	2014	20,964	7861	3581	3402
Comoros	2018	3149	1259	1253	1129
Liberia	2014	7606	1499	1310	1281
Lesotho	2014	3138	655	640	204
Malawi	2013	17,286	2207	1990	1878
Mali	2016	10,326	1960	2214	2138
Niger	2018	12,558	2283	2442	2334
Namibia	2013	5046	952	861	819
Rwanda	2012	7856	1524	1580	1526
Sierra Leone	2015	11,938	2150	2204	2108
Senegal	2017	12,326	1908	1764	1710
Chad	2013	18,623	4217	4287	4052
Togo	2016	6979	1395	874	838
Tanzania	2014	10,233	4163	4123	3964
Uganda	2016	15,522	1954	1924	1811
Zambia	2018	13,457	5043	2959	2793
Zimbabwe	2015	6132	2240	2355	2293
Total		316,600	74,984	62,992	60,038

^a^All children under-five; ^b^children under 2 years; ^c^children whose mothers had information on breastfeeding; ^d^children whose mothers had complete information on all variables of interest

### Definition of variables

#### Outcome variable

The outcome variable for the study was timely initiation of breastfeeding. It was derived from the question, “How long after birth did you first put (NAME) to the breast?” The responses were: immediately, hours and days [18, 19]. The responses were then dichotomised to timely initiation of breastfeeding = 1, if women responded that breastfeeding was done immediately after birth or within the first hour, and late initiation = 0, if women responded otherwise [18, 19].

#### Independent variables

The study used seventeen independent variables. These variables were considered principally because of their statistically significant relationship with timely initiation of breastfeeding in previous studies [12–14, 18, 19]. These variables were grouped into maternal factors child factors and contextual factor. The maternal factors included mother’s age, mother's educational level, marital status, employment status, frequency of reading newspaper, frequency of listening to radio, frequency of watching television, number of antenatal care (ANC) visits, place of residence, wealth quintile, type of delivery assistance, place child was delivered, and type of delivery. Wealth quintile is computed based on household assets and characteristics of the household. In DHS, wealth quintile is computed using Principal Component Analysis (PCA). The child factors used for the study were size of the child at birth, birth order and twin status of the child (Table 2). The contextual factor was sub-region and was grouped into West Africa, South Africa, Central Africa, and East Africa.

**Table 2:** Distribution of timely initiation of breastfeeding in SSA by explanatory variables (Weighted, *N* = 60,038)

Variables	Weighted ***N***	Weighted %	Timely initiation of breastfeeding	***P*** - value
**Mother's age**				90.4 (< 0.001)
15–19	6122	10.2	50.6	
20–24	14,954	24.9	55.4	
25–29	16,200	27.0	57.1	
30–34	11,723	19.5	57.1	
35–39	7439	12.4	56.6	
40–44	2953	4.9	56.7	
45–49	647	1.1	57.8	
**Marital status**				16.5 (0.002)
Never married	3745	6.2	55.8	
Married	443,388	73.9	56.3	
Cohabiting	8670	14.4	54.0	
Widowed	539	0.9	58.4	
Divorced	2695	4.5	56.2	
**Mother’s educational level**				163.6 (< 0.001)
No education	25,399	42.3	53.1	
Primary	21,283	35.5	59.0	
Secondary+	13,355	24.2	56.6	
**Employment status**				24.5 (< 0.001)
Not working	20,958	34.9	57.3	
Working	39,080	65.1	55.2	
**Place of residence**				1.7 (0.196)
Urban	15,854	26.4	55.5	
Rural	44,184	73.6	56.1	
**Wealth quintile**				52.5 (< 0.001)
Poorest	14,547	24.2	54.3	
Poorer	13,394	22.3	55.0	
Middle	12,079	20.1	56.3	
Richer	10,949	18.2	56.9	
Richest	9069	15.11	58.7	
**Frequency of reading newspaper**				27.8 (< 0.001)
Not at all	51,638	86.0	55.5	
Less than once a week	5206	8.7	59.3	
At least once a week	3194	5.3	57.1	
**Frequency of listening to radio**				22.5 (< 0.001)
Not at all	26,982	44.9	55.3	
Less than once a week	12,055	20.1	55.1	
At least once a week	21,000	35.0	57.3	
**Frequency of watching television**				26.8 (< 0.001)
Not at all	41,527	69.2	56.6	
Less than once a week	7202	12.0	54.5	
At least once a week	11,309	18.8	54.2	
**Number of ANC visits**				214.0 (< 0.001)
0	6126	10.2	47.5	
1–3	21,951	36.6	56.1	
4+	31,961	53.2	57.5	
**Size of child at birth**				183.3 (< 0.001)
Larger than average	20,636	34.4	54.3	
Average	28,955	48.2	58.6	
Smaller than average	10,446	17.4	51.7	
**Birth order**				100.4 (< 0.001)
1st	12,563	20.9	53.2	
2–4	28,640	47.7	58.0	
5+	18,835	31.4	54.6	
**Place of delivery**				701.3 (< 0.001)
Home	20,329	33.9	48.5	
Health facility	39,709	66.1	59.8	
**Assisted by a health worker**				171.9 (< 0.001)
No	26,164	41.9	49.6	
Yes	34,874	53.1	60.6	
**Type of delivery**				569.8 (< 0.001)
Vaginal birth	56,987	94.9	57.0	
Cesarean section	3051	5.1	34.6	
**Twin status**				49.3 (< 0.001)
Single birth	59,028	98.3	56.1	
Multiple birth	1010	1.7	45.1	

### Statistical analyses

The data were analysed with Stata version 14.0 (Stata Corporation, College Station, TX, USA). The analyses were done in three steps. The first step was the computation of the prevalence of timely initiation of breastfeeding in SSA. The second step was a bivariate analysis that calculated the proportions of timely initiation of breastfeeding across the socio-demographic characteristics with their significance levels. After the bivariate analysis, we checked for high correlation among the significant explanatory variables through a test for multicollinearity using the variance inflation factor (VIF) and the results showed no evidence of high collinearity (Mean VIF = 1.47, Maximum VIF = 2.76, and Minimum VIF = 1.02). Afterwards, three hierarchical logistic regression models were built. Only variables that were significant from the second step were used in a multivariable logistic regression (Table 3). Model I constituted a multivariable analysis between the maternal factors and timely initiation of breastfeeding. We added child factors to the initial model in Model II. Model III controlled for the effect of sub-regions. We presented all results of the logistic analyses as adjusted odds ratios (aORs) with 95% confidence intervals (CIs). All frequency distributions were weighted using the sample weight (v005/1,000,000) whiles the svy command was used to account for the complex survey design and generalizability of the findings.

**Table 3:** Multivariable hierarchical logistic regression analysis on determinants of timely initiation of breastfeeding in SSA

Variable	Model I aOR 95% CI	Model II aOR 95% CI	Model III aOR 95% CI
**Mother's age**			
15–19	1	1	1
20–24	1.26^***^ (1.18,1.34)	1.20^***^ (1.13,1.28)	1.18^***^ (1.10,1.26)
25–29	1.40^***^ (1.32,1.49)	1.35^***^ (1.26,1.46)	1.32^***^ (1.22,1.42)
30–34	1.41^***^ (1.32,1.51)	1.45^***^ (1.34,1.58)	1.38^***^ (1.27,1.50)
35–39	1.41^***^ (1.31,151)	1.51^***^ (1.38,1.65)	1.43^***^ (1.30,1.56)
40–44	1.44^***^ (1.31,157)	1.56^***^ (1.40,1.74)	1.49^***^ (1.33,1.66)
45–49	1.56^***^ (1.33,1.84)	1.71^***^ (1.44,2.04)	1.63^***^ (1.36,1.94)
**Wealth quintile**			
Poorest	1	1	1
Poorer	0.97 (0.93,1.02)	0.97 (0.921,1.01)	0.99 (0.94,1.03)
Middle	1.01 (0.96,1.06)	1.01 (0.96,1.06)	1.02 (0.97,1.07)
Richer	1.03 (0.98,1.09)	1.02 (0.97,1.08)	1.02 (0.97,1.08)
Richest	1.19^***^ (1.011,1.27)	1.17^***^ (1.10,1.25)	1.13^***^ (1.05,1.20)
**Marital status**			
Never married	1	1	1
Married	0.98 (0.91,1.06)	0.96 (0.89,1.03)	0.91^*^ (0.85,0.98)
Cohabiting	0.86^***^ (0.79,0.93)	0.84^***^ (0.78,0.91)	0.87^**^ (0.80,0.95)
Widowed	1.04 (0.87,1.25)	1.02 (0.84,1.22)	1.00 (0.83,1.20)
Divorced	0.94 (0.85,1.05)	0.92 (0.83,1.02)	0.90^*^ (0.81,1.00)
**Mother’s education level**			
No education	1	1	1
Primary	1.22^***^ (1.17,1.27)	1.21^***^ (1.16,1.26)	1.21^***^ (1.16,1.26)
Secondary+	1.03 (0.97,1.08)	1.02 (0.96–1.07)	1.03 (0.98,1.09)
**Employment status**			
Not working	1	1	1
Working	0.85^***^ (0.82,0.88)	0.85^***^ (0.82,0.88)	0.90^***^ (0.87,0.93)
**Frequency of reading newspaper**			
Not at all	1	1	1
Less than once a week	1.14^***^(1.07,1.22)	1.13^***^ (1.06,1.21)	1.13^***^ (1.06,1.21)
At least once a week	1.07 (0.98,1.16)	1.06 (0.97,1.15)	1.09^*^ (1.00,1.19)
**Frequency of listening to radio**			
Not at all	1	1	1
Less than once a week	0.96 (0.92,1.01)	0.96 (0.92,1.01)	0.93^**^ (0.88,0.97)
At least once a week	1.07^***^ (1.02,1.11)	1.06^**^ (1.02,1.10)	1.03 (0.99,1.07)
**Frequency of watching to television**			
Not at all	1	1	1
Less than once a week	0.81^***^ (0.77,0.87)	0.82^***^ (0.78,0.87)	0.82^***^ (0.77,0.86)
At least once a week	0.71^***^ (0.67,0.75)	0.70^***^ (0.67,0.74)	0.74^***^ (0.70,0.78)
**Number of ANC visits**			
0	1	1	1
1–3	1.13^***^ (1.07,1.20)	1.12^***^ (1.05,1.19)	1.10^**^ (1.03,1.17)
4+	1.15^***^ (1.08,1.22)	1.13^***^ (1.07,1.20)	1.11^**^ (1.03,1.17)
**Place of delivery**			
Home	1	1	1
Health facility	1.32^***^ (1.24,1.39	1.29^***^ (1.22,1.37)	1.41^***^ (1.33,1.50)
**Assisted by a health worker during delivery**			
No	1	1	1
Yes	1.39^***^ (1.32,1.47)	1.41^***^ (1.34,1.49)	1.26^***^ (1.19,1.33)
**Type of delivery**			
Vaginal birth	1	1	1
Cesarean section	0.31^***^ (0.28,0.33)	0.31^***^ (0.29,0.34)	0.30^***^ (0.27,0.32)
**Size of child at birth**			
Larger than average		1	1
Average		1.20^***^ (1.15,1.24)	1.19^***^ (1.15,1.24)
Smaller than average		0.97 (0.92,1.02)	0.94^*^ (0.90,0.99)
**Birth order**			
1st		1	1
2–4		1.13^***^ (1.07,1.19)	1.15^***^ (1.09,1.21)
5+		0.93^*^ (0.87,1.00)	0.97 (0.90,1.04)
**Twin status**			
Single birth		1	1
Multiple birth		0.65^***^ (0.57,0.74)	0.67^***^ (0.59,0.76)
**Sub Region**			
West Africa			1
East Africa			1.71^***^ (1.64,1.78)
Central Africa			0.80^***^ (0.75,0.84)
Southern African			2.13^***^ (1.97,2.31)
**N**	60,038	60,038	60,038
**Pseudo R**^**2**^	0.027	0.030	0.046

Exponentiated coefficients; 95% confidence intervals in brackets

^*^ *p* < 0.05, ^**^ *p* < 0.01, ^***^ *p* < 0.001

*aOR* Adjusted Odds Ratio, *CI* Confidence Interval

Model I: Maternal factors

Model II: Child factors

Model III: Maternal factors, child factors and sub-region

## Results

### Prevalence of timely initiation of breastfeeding in sub-Saharan Africa

**Fig. 1:**
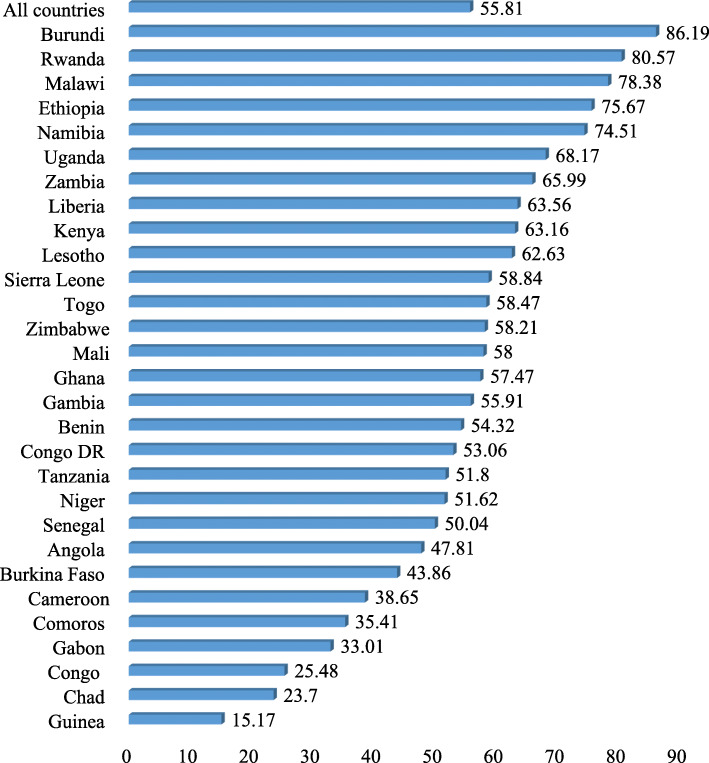
Proportion of women who engaged in timely initiation of breastfeeding

Figure 1 presents results on the prevalence of timely initiation of breastfeeding in SSA. We found a prevalence of 55.81% in the sub-region. The country with the highest prevalence of timely initiation of breastfeeding was Burundi (86.19%), whereas Guinea had the lowest prevalence (15.17%).

### Bivariate results on the determinants of timely initiation of breastfeeding

Table 2 presents results of the distribution of timely initiation of breastfeeding in SSA accross the explanatory variables. All the explanatory variables, except place of residence had significant associations with timely initiation of breastfeeding at 95% CI. The highest prevalence of timely initiation of breastfeeding was among women aged 45–49 (57.8%), those who were widowed (58.4%), women with primary education (59.0%), non-working women (57.3%), those with richest wealth quintile (58.7%), those who read newspaper less than once a week (59.3%), those who listened to radio at least once a week (57.3%), and those who never watched television (56.6%). Women who had four or more ANC visits (57.5%), those with average sized children (58.6%), those with 2–4 birth order children (58.0%), those who delivered in the health facility (59.8%), those who had assistance of a health professional during delivery (60.6%), those who had vaginal delivery (57.0%), and those with single births (56.1%), all had high prevalence of timely initiation of breastfeeding.

### Results of the multivariable logistic regression analysis

Table 3 presents results of the multivariable hierarchical logistic regression analysis on the maternal and child factors associated with timely initiation of breastfeeding in SSA. Model III, which is the complete model, presents the results for all the determinants, while controlling for sub-regions. The results show that the likelihood of timely initiation of breastfeeding was higher among women of all age categories compared with those aged 15–19, with the highest odds among those aged 45–49 (aOR 1.63; 95% CI 1.36, 1.94). Richest women were more likely to practice timely initiation of breastfeeding compared to women with poorest wealth quintile (aOR 1.13; 95% CI 1.05, 1.20). Higher odds of timely initiation of breastfeeding were also observed among women with primary education (aOR 1.21; 95% CI 1.16, 1.26), those who read newspaper at least once a week (aOR 1.13; 95% CI 1.06, 1.21), women who had four or more ANC visits (aOR 1.11; 95% CI 1.03, 1.17) and women who delivered at the health facility (aOR 1.41; 95% CI 1.33, 1.50), compared to women with no formal education, those who never read newspaper, those with no ANC visits and those who delivered at home. Women who were assisted by a health professional during delivery, compared to those who were not (aOR 1.26; 95% CI 1.19, 1.33), those who had 2–4 birth order children compared to those with first birth order children (aOR 1.15; 95% CI 1.09, 1.21) and women who lived in Southern Africa compared to those who lived in West Africa (aOR 2.13; 95% CI 1.97, 2.31) were more likely to initiate breastfeeding timely.

Lower odds of timely initiation of breastfeeding were identified in married women, compared to never married women (aOR 0.91; 95% CI 0.85, 0.98); working women, compared to non-working women (aOR 0.90; 95% CI 0.87, 0.93); women who listened to radio less than once a week, compared to those who never listened to radio (aOR 0.93; 95% CI 0.88, 0.97); women who watched television at least once a week, compared to those who never watched television (aOR 0.74; 95% CI 0.70, 0.78); women who delivered through caesarean section, compared to those who had vaginal delivery (aOR 0.30; 95% CI 0.27, 0.32); women who had smaller than average children compared to those with larger than average children (aOR 0.94; 95% CI 0.90, 0.99); and those with multiple births, compared to those with single births (aOR 0.67; 95% CI 0.59, 0.76). Women who lived in Central Africa were less likely to initiate breastfeeding timely compared to those who lived in West Africa (aOR 0.80; 95% CI 0.75, 0.84). 

## Discussion

The study sought to examine the maternal and child factors associated with timely initiation of breastfeeding in SSA. The results indicated that timely initiation of breastfeeding was phenomenal among Southern and East Africans. A meta-analysis conducted by Issaka and colleagues about the prevalence of key breastfeeding indicators in 29 sub-Saharan African countries similarly found that women in Southern Africa were inclined to timely initiation of breastfeeding [12]. Hence, underlying factors influencing mothers’ timely initiation of breastfeeding practices across SSA need a critical attention. Meanwhile, a review by Bora [20] noted that educational interventions significantly increased breastfeeding rates in developing countries. Therefore, similar interventions could aid improve maternal knowledge and practices of timely initiation of breastfeeding among women in SSA, especially residents in Central Africa. In relation to the above, the present study identified specific maternal and child factors associated with timely initiation of breastfeeding.

The study revealed that maternal socio-economic status was positively associated with timely initiation of breastfeeding. The results are consistent with a systematic review of literature on factors and barriers accounting for timely initiation of breastfeeding in South Asia [21]. Also, birth order correlated with timely initiation of breastfeeding whereby women at parity 2–4 had higher odds to initiate breastfeeding timely. Probably, women of higher parity might be refining their knowledge and practices about breastfeeding with each birth [1], hence, affecting their breastfeeding practices. Consistent with a previous study [9], timely initiation of breastfeeding correlated with women who delivered at the health facility. Similarly, the study revealed a positive association between health worker’s assisted delivery and timely initiation of breastfeeding. This is not surprising since mothers who delivered in the health facilities can benefit from direct counselling provided by health professionals on the practice of timely initiation of breastfeeding [10].

In disagreement to a previous study [9], the prevailing study observed a negative association between mother’s level of education and timely initiation of breastfeeding. Arguably, education propels women to be receptive to health information which refines their behavior and reorient them to select positive health behaviours, including adopting healthy infant feeding practices such as timely initiation of breastfeeding [22], so our observation was unexpected. Moreover, an inverse relationship was noted between access to radio and timely initiation of breastfeeding. Theoretically, having access to information through radio might not necessarily translate into usage of such information if the information is culturally incompatible and complex to adopt [23]. Other maternal factors that determined timely initiation of breastfeeding include marital status and ANC visits. Although, a study conducted across nine countries in SSA had reported that when mothers attended ANC frequently, their likelihood to timely initiate breastfeeding increased [24]. However, the cross-sectional nature of the present study limited the effort to explain the reasons accounting for this relationship. Therefore, further studies to explain the relationship between these maternal variables and timely initiation of breastfeeding will be beneficial.

The study noted an inverse association between child’s birthweight and timely initiation of breastfeeding, whereby decreasing odds to timely initiate breastfeeding was found among women with children smaller than average size. Plausibly, some of the children born underweight may be undergoing the continuous maturing process of the physiologic functions relating to ‘nutritive sucking pathway’ development [25]. Hence, limiting their abilities needed for breastfeeding within the first hour of life, including having a good coordination of the suction-deglutition respiration cycle and the breast-seeking reflex [26]. Delivering through caesarean section was negatively associated with timely initiation of breastfeeding. Yisma et al. in their meta-analysis about impact of caesarean section on breastfeeding indicators among 33 countries in sub-Saharan Africa, showed that caesarean section was associated with a 46% lower prevalence of timely initiation of breastfeeding [14]. It is argued that mothers undergo a lot of pain due to caesarean delivery which contribute to the delay in breastfeeding practice [26]. Other literature also suggest that effects of anaesthesia could delay the onset of lactation or babies delivered through caesarean section faces associated respiratory distress [1]. Finally, the study revealed an inverse association between multiple births and timely initiation of breastfeeding. It is known that mothers who deliver multiple babies often experience difficulty with the initiation of breastfeeding during their hospitalisation [27]. Furthermore, the birth of twin babies is physically and mentally demanding compared to a singleton birth and is associated with numerous obstacles to breastfeeding [28].

### Strengths and limitations

The novelty and strength linked with this study stem from the fact that, it investigated both maternal and child factors associated with timely initiation of breastfeeding in SSA using a nationally representative survey datasets from 29 countries. Also, the probability method employed in selecting survey respondents matched with appropriate analytical procedure make the results of the study robust. Again, the two-stage sampling approach used ensured that there was no selection bias that could affect the results. However, our results should be interpreted with caution. Firstly, causality cannot be established due to the cross-sectional nature of the study. Again, some variables had missing data that were treated as complete cases. Notwithstanding, the final sample size used, had a higher response rate which masked the effect of missing data. Also, the time interval between delivery and the interview up to 5 years can lead to recall bias which can result in inaccurate responses from mothers regarding the timeframe they put their babies to breastmilk. Additionally, surveyed women might provide responses concerning the practices of timely initiation of breastfeeding with the view of creating a positive breastfeeding image for themselves among those who know it is desirable, hence, social desirability bias is unavoidable in this study. Again, the interpretation of the odds should be done with caution as we admit that the higher odds ratios obtained in relation with some of the variables could be due to the large sample size used in this study. Moreover, the percentage variance in the models could have been increased by the inclusion of some sociocultural variables like colonial past, which were not available in the datasets. Finally, we admit that our study design did not permit us to investigate the reasons why disparities occurred, as far as timely initiation of breastfeeding and maternal and child factors are concerned.

## Conclusions

The maternal factors identified to influence timely initiation of breastfeeding were mother’s wealth quintile, educational attainment, employment status, type of delivery assistance, place of delivery, type of delivery, marital status, ANC visits, and country of residence. The associated child factors were child’s size at birth, birth order and twin status. It is recommended that prioritising policies to enhance timely initiation of breastfeeding is needed, particularly among Central African countries where timely initiation of breastfeeding remains a challenge. Sufficient supportive care in addition to guidance and counselling should be given to mothers, especially those who go through caesarean section, deliver children smaller than average size or encounter multiple births to resolve timely initiation of breastfeeding inequalities associated with such class of mothers. Behavioural change communication programmes targeted at timely initiation of breastfeeding to reverse and close the timely initiation of breastfeeding gaps stratified by the maternal and child factors is worth implemented in SSA.

## Data Availability

The dataset is freely available for download at: https://dhsprogram.com/data/available-datasets.cfm.

## Abbreviations

ANC: Antenatal Care
CI: Confidence interval
CS: Caesarean section
DHS: Demographic and Health Surveys
EA: Enumeration areas
GSIYF: Global Strategy for Infant and Young Child Feeding
SSA: Sub-Saharan Africa
STROBE: Strengthening the Reporting of Observational Studies in Epidemiology
VIF: Variance inflation factor
WHO: World Health Organization
